# Influence of Tapered‐End and Round‐End Bristle Designs on the Abrasive Enamel Wear Using Toothpastes With High Discrepancies in Abrasiveness

**DOI:** 10.1111/idh.70073

**Published:** 2026-04-23

**Authors:** Michelle S. Stübi, Moritz Tanner, Blend Hamza, Leonardo Svellenti, Thomas Attin, Florian J. Wegehaupt

**Affiliations:** ^1^ Postgraduate Student, Clinic of Conservative and Preventive Dentistry, Center for Dental Medicine University of Zürich Zürich Switzerland; ^2^ Resident Physician, Clinic of Conservative and Preventive Dentistry, Center for Dental Medicine University of Zürich Zürich Switzerland; ^3^ Resident Physician, Clinic of Orthodontics and Pediatric Dentistry, Center for Dental Medicine University of Zürich Zürich Switzerland; ^4^ Resident Physician, Department of Periodontology, Endodontology and Cariology University Center for Dental Medicine Basel UZB, University of Basel Basel Switzerland; ^5^ Professor and Director, Clinic of Conservative and Preventive Dentistry, Center for Dental Medicine University of Zürich Zürich Switzerland; ^6^ Professor and Head Division of Preventive Dentistry and Oral Epidemiology, Clinic of Conservative and Preventive Dentistry, Center for Dental Medicine University of Zürich Zürich Switzerland

**Keywords:** abrasion, bristle, dentistry, enamel, REA, toothbrush

## Abstract

**Purpose:**

The aim of this in vitro study was to investigate the influence of bristle end design of toothbrushes and the relative enamel abrasivity (REA) of used toothpaste on the resulting abrasive enamel wear.

**Materials and Methods:**

A total of 160 bovine enamel samples were allocated into eight groups (*n* = 20) and surface topography was recorded profilometrically. Samples of groups A–D were brushed with a toothbrush with a tapered‐end bristle design, and samples of groups E–H with a round‐end bristle design. Brushing was performed in an automatic brushing machine with a frequency of 60 brushing cycles per minute, with reciprocating movements at a constant brushing force of 2.5 N for a total of 6 h. In groups A and E, toothpaste Colgate Total (CT; REA 4); in groups B and F, toothpaste Candida White Diamond (CWD; REA 244); in groups C and G, toothpaste Swiss Smile Diamond Glow (SSDG; REA 177); and in groups D and H, toothpaste Emoform F Diamond (EFD; REA 51) were used. Surface topography was recorded after 6 h of brushing again, and the abrasive enamel wear was calculated.

**Results:**

For all three diamond‐based toothpastes (CWD, SSDG and EFD), brushing with the round‐end toothbrush caused statistically significantly more wear than with the tapered‐end toothbrush (*p* < 0.05). For both toothbrushes, SSDG caused the highest abrasive wear (51.36/6.34 μm resp. 29.71/15.35 μm), followed by CWD (47.61/18.66 μm resp. 7.16/2.23 μm) and EFD (16.91/2.86 μm resp. 5.27/2.11 μm), with the respective differences being statistically significant (median/interquartile range IQR).

**Conclusion:**

In combination with diamond‐based toothpastes, tapered‐end toothbrushes cause less abrasive enamel wear compared to round‐end toothbrushes.

## 1 Introduction

Achieving and maintaining satisfactory oral health requires appropriate knowledge, tools, and techniques. Brushing teeth with a toothbrush is a simple, practical, and probably the most common way to achieve this goal and is often combined with the use of toothpaste.

With their origins going back several millennia, today, mainly acrylic‐based manual toothbrushes of various configurations are used. Accordingly, there have already been various studies comparing these different types of toothbrushes and their effects on different clinically relevant parameters. For standardization, the American Dental Association (ADA) defined a reference toothbrush with a flat bristle field, parallel arranged cylindrical filaments and rounded ends. Especially, the round end design of the filaments was considered an important quality feature at the time, as they were repeatedly proved to cause less gingival abrasion and injuries compared to previously widely used sharp‐cut bristles [1, 2, 3, 4, 5, 6, 7, 8, 9, 10, 11, 12, 13, 14, 15, 16, 17, 18, 19, 20, 21, 22, 23, 24, 25, 26, 27, 28, 29, 30, 31, 32, 33, 34, 35].

However, since then, numerous in vitro and in vivo studies showed superiority of toothbrush configurations different to the one described by the ADA in terms of specific parameters such as plaque removal or reduction of various gingival indices indicating the degree of inflammation or abrasion [10, 11, 12, 13, 14, 15, 16, 17, 18, 19, 20, 21, 22, 23, 24, 25, 26, 27, 28, 29, 30, 31, 32, 33, 34, 35, 36, 37, 38, 39, 40, 41, 42, 43, 44, 45, 46, 47, 48, 49, 50, 51, 52].

At the beginning of the 2000s, a new type of toothbrush for periodontal purposes was developed. Instead of the traditional round‐end design, its brush head was provided with conical filaments that become thinner towards the tip. In contrast to round‐ended toothbrushes, which are usually manufactured using sanding paper for bristle trimming, toothbrushes with a tapered end are given their final shape by drawing them up in an acid solution, which causes the bristles to become continuously thinner towards the tip. The idea was that the slim design and the resulting higher bending capacity at the tips would make it easier to clean hard to reach places such as interdental spaces or gingival margins. And indeed, a series of in vitro studies performed by Yankell et al. showed toothbrushes with conical filaments to be significantly more effective when it comes to cleaning efficacy in both interproximal as well as marginal and subgingival sites compared to the ADA reference toothbrush with its round‐end bristles [5, 6, 7, 8, 9, 10, 11, 12, 13, 14, 15, 16, 17, 18, 19, 20, 21, 22, 23, 24, 25, 26, 27, 28, 29, 30, 31, 32, 33, 34, 35, 36, 37, 38, 39, 40, 41, 42, 43, 44, 45, 46, 47, 48, 49, 50, 51, 52, 53, 54].

Further in vivo studies showed a significant improvement of plaque‐related gingival indices [37, 38, 39] as well as a reduction in gingival abrasion scores [11] for the tapered‐end toothbrushes. A more recent systematic review by Ranzan et al. [33] confirmed this finding, even though the differences in gingival abrasion were only minor and with the stiffness of the bristles being more decisive. In addition, tapered‐end toothbrushes are becoming increasingly popular among consumers, as a majority perceived them to be more pleasant to use and subjectively to clean better [36, 37, 38, 39].

However, despite these aforementioned advantages of tapered‐end filaments in terms of clinical parameters, the current literature also contains references to disadvantageous properties such as more distinct abrasive wear of dental hard tissues. It has to be mentioned that—beside other factors such as frequency, technique and brushing force [1, 2, 3, 4, 5, 6, 7, 8, 9, 10, 11, 12, 13, 14, 15, 16, 17, 18, 19, 20, 21, 22, 23, 24, 25, 26, 27, 28, 29, 30, 31, 32, 33, 34, 35, 36, 37, 38, 39, 40, 41, 42, 43, 44, 45, 46, 47, 48, 49, 55, 56] the use of toothpaste and its abrasiveness plays a major role in abrasive tooth wear, with partially different behaviour on enamel and dentine depending on the type of abrasive particles used [1, 2, 3, 4, 5, 6, 7, 8, 9, 10, 11, 12, 13, 14, 15, 16, 17, 56]. The increase in tooth wear is attributed to the higher flexibility of the conical, tapered‐end filaments. Their correspondingly lower stiffness results in greater bending and therefore in a larger contact area, thus enabling not only more efficient plaque removal, but also more abrasive wear [6, 7, 8, 9, 10, 11, 12, 13, 14, 15, 16, 17, 18, 19, 20, 21, 22, 23, 24, 25, 26, 27, 28, 29, 30, 31, 32, 33, 34, 35, 36, 37, 38, 39, 40, 41, 42, 43, 44].

However, most of the studies performed on this topic used toothbrushes with partially great differences in their specifications such as material, diameter, length, and stiffness of the filaments used or their configuration on the brushing head [7, 8, 9, 10, 11, 12, 13, 14, 15, 16, 17, 18, 19]. For comparability, it is essential that all parameters are kept identical to the biggest extent possible, except for the one of particular interest.

A recent study by Hamza et al. [19] compared two types of toothbrushes that differed only in their bristle end configuration (tapered‐end vs. round‐end), with all the other specifications being identical. The tapered‐end toothbrush was shown to cause statistically significantly less abrasive dentine wear compared to the round‐end toothbrush, regardless of the applied brushing force (1 N–4 N).

However, this has not yet been investigated on enamel. It is known from numerous previous in vitro abrasion studies that the corresponding results on enamel and dentine can differ greatly [9, 10, 11, 12, 13, 14, 15, 16, 17, 18, 19, 20, 21, 22, 23, 24, 25, 26, 27, 28, 29, 30, 31, 32, 33, 34, 35, 36, 37, 38, 39, 40, 41, 42, 43, 44, 45, 46]. This may be partly explained by the different composition and structure of the two types of dental hard tissue [21, 22, 23, 24, 25, 26, 27, 28, 29, 30, 31]. In addition, there appear to be other decisive factors which may also have an impact on the outcome, including the design of the study and various experimental factors influencing the abrasion process. It has been shown that the use of toothbrushes and, in particular, their interplay with toothpaste seems to have a major impact on abrasive tooth wear in general [1, 2, 3, 4, 5, 6, 7, 8, 9, 10, 11, 12, 13, 14, 15, 16, 17, 18, 19, 20, 21, 22, 23, 24, 25, 26, 27, 28, 29, 30, 31, 32, 33, 34, 35, 36, 37, 38, 39, 40, 41, 42, 43, 44, 45, 46, 47, 48, 49, 56].

The degree of abrasiveness of toothpastes is often indicated by the relative dentine abrasivity value (RDA). But in healthy patients, abrasive wear caused by oral health measurements usually takes place on enamel. It would therefore make more sense to specify the less common REA. Furthermore, although they may provide an indication of abrasiveness, RDA as well as REA values were shown not necessarily to correlate with one another nor with the effective, absolute removal of tooth substances [28, 29, 30, 31, 32, 33, 34, 35, 36, 37, 38, 39, 40, 41].

With the introduction of new abrasives in toothpastes such as diamond powder, some fundamental assumptions regarding the abrasivity behaviour need to be reconsidered. In contrast to traditionally used silicate particles, a recent study by Hamza et al. revealed some diamond powder‐based toothpastes to exhibit relatively low RDA values while at the same time comparably high REA values [17]. A further study also emphasized their different behaviour on enamel and dentine, whereby too, REA values given did not allow direct conclusions to be drawn about the quantitative removal of enamel [16].

Therefore, the aim of the present study was to investigate the effect of bristle end designs (tapered‐end and round‐end) on the abrasive enamel wear using toothpastes of different abrasivity. The following two null hypotheses were tested: [55] there are no differences in enamel wear when brushing with tapered‐end and round‐end toothbrushes and [56] toothpastes with widely differing REA values show no differences in the absolute abrasive enamel wear.

## 2 Materials and Methods

### 2.1 Sample Preparation and Allocation

For this study, 160 enamel samples from 40 lower bovine incisors from slaughterhouse waste were used. Sample size was selected based on empirical values from previous studies. Only teeth without enamel grooves and enamel cracks from which four samples could be obtained were used. Samples were gained from the buccal crown surface using a diamond‐coated trephine drill with an inner diameter of 3 mm (Proxxon bench drill, Brütsch/Rüegger Werkzeuge AG, Urdorf, Switzerland). Per single tooth four samples were gained. From teeth 1–20, samples A–D were gained and from teeth 21–40, samples E–H were gained. Samples were then embedded in acrylic resin (Paladur, Kulzer GmbH, Hanau, Germany) using a silicone cast with an inner diameter of 6 mm and a lateral notch, thus leaving only the surface of enamel uncovered. The acrylic resin was polymerized in a laboratory curing unit (Palamat Elite, Kulzer GmbH, Hanau, Germany) at 45°C at 3 bar air pressure for 10 min. An automatic grinding machine (Tegramin‐30, Struers GmbH, Willich, Germany) was used to polish the surfaces with grinding papers of decreasing roughness (waterproof silicon carbide paper #1200, #2000 and #4000; Struers GmbH, Willich, Germany) under constant water cooling at a contact pressure of 5 N for 10 s, 20 s and 30 s, respectively. Samples were then reduced from the backside (composed of embedding resin) with a metal drill (Proxxon bench drill, Brütsch/Rüegger Werkzeuge AG, Urdorf, Switzerland) to a final thickness of 3 mm and stored in tap water at 5°C.

Samples were allocated into eight groups (*n* = 20). All samples A were allocated in group A, all samples B in group B, and so on.

### 2.2 Brushing Procedure

Groups A–D were used to test toothbrushes with tapered‐end bristle design and groups E–H with round‐end bristle design. The exact toothbrush specifications used can be found in Table 1. The samples of groups A and E served as control groups and were brushed with Colgate Total Original (GABA Schweiz AG, Therwil, Switzerland), a silicate‐based toothpaste. The remaining groups were brushed with toothpastes containing diamond particles. Samples of groups B and F were brushed with Candida White Diamond (Mibelle AG, Buchs AG, Switzerland), C and G with Swiss Smile Diamond Glow (Curaden AG, Kriens, Switzerland), and D and H with Emoform F Diamond (Dr. Wild & Co. AG, Muttenz, Switzerland). Group allocation of samples and experimental design are illustrated in Table 2.

**TABLE 1:** Toothbrush specifications according to the manufacturers.

Bristle end configuration	Round‐end	Tapered‐end
Bristle length	11.5 ± 0.2 mm	12 ± 0.2 mm
Bristle diameter	7 mils	7 mils
Bristle material	Polyethylenterephthalate (PET)	Polyethylenterephthalate (PET)
Number of tufts	35	35
Number of bristles per tuft	48 ± 4	48 ± 4

**TABLE 2:** Group allocation of samples and experimental design.

**Preparation of 160 enamel samples from 40 bovine incisors** (Samples A–D from teeth 1–20; Samples E–H from teeth 21–40)							
**Profilometer (baseline measurement)**							
**Group**							
A	B	C	D	E	F	G	H
*n* = 20	*n* = 20	*n* = 20	*n* = 20	*n* = 20	*n* = 20	*n* = 20	*n* = 20
Samples A	Samples B	Samples C	Samples D	Samples E	Samples F	Samples G	Samples H
**Group‐specific brushing of the samples (automatic brushing machine)** (60 brushing cycles per min; a total of 21,600 brushing cycles per sample; slurry 1:2; change slurry every 5400 brushing cycles)							
**Bristle end configuration**							
**Tapered‐end**				**Round‐end**			
**Toothpaste**							
Colgate Total	Candida White	Swiss Smile Diamond	Emoform F Diamond	Colgate Total	Candida White	Swiss Smile Diamond	Emoform F Diamond
Original (REA 4)	Diamond (REA 244)	Glow (REA 177)	(REA 51)	Original (REA 4)	Diamond (REA 244)	Glow (REA 177)	(REA 51)
**Profilometer (final measurement)**							

Previously measured REA and RDA values, ingredients and type of abrasives of the toothpastes used are shown in Table 3.

**TABLE 3:** Composition of the tested toothpastes according to the manufacturers.

Toothpaste	REA*	RDA*	Ingredients	Abrasives
Colgate Total Original (GABA Schweiz AG, Therwil, Switzerland)	4	100	Glycerin, Aqua, Hydrated silica, Sodium lauryl sulfate, Arginine, Aroma, Cellulose gum, Zinc oxide, Poloxamer 407, Zinc citrate, Tetrasodium Pyrophosphate, Xanthan gum, Benzyl alcohol, Cocamidopropyl betaine, Sodium fluoride (1′450 ppm), Sodium saccharin, Phosphoric acid, Sucralose, CI 77891	Hydrated silica
Candida White Diamond (Mibelle AG, Buchs AG, Switzerland)	244	12	Aqua, Hydrogenated starch Hydrolysate, Potassium citrate, Hydrated silica, Sodium lauryl sulfate, Xanthan gum, Aroma, Sodium acrylates/C10‐30 Alkyl acrylate Crosspolymer, Sodium fluoride (1′450 ppm), Sodium saccharine, Zinc chloride, Diamond powder, Methylparaben, Allantoin, Limonene, Linalool, Benzyl alcohol, CI 77891.	Hydrated silica, Diamond powder
Swiss Smile Diamond Glow (Curaden AG, Kriens, Switzerland)	177	14	Aqua, Sorbitol, Glycerin, Hydrated silica, Titanium dioxide, Bentonite, Hydroxyapatite, Decyl glucoside, Mica, Aroma, Sodium monofluorophosphate (980 ppm), Cocamidopropyl betaine, Tocopherol, Xanthan gum, Glucose oxidase, Potassium chloride, Maltodextrin, Diamond powder, Sodium saccharin, Curcuma xanthorrhiza Root extract, *Aloe barbadensis* juice extract, Sodium hydroxide, Sodium benzoate, Tin oxide, Silica, D‐Limonene, Linalool, Eugenol	Hydrated silica, Hydroxylapatite, Diamond powder, Silica
Emoform F Diamond (Dr. Wild & Co. AG, Muttenz, Switzerland)	51	42	Glycerin, Silica, Aqua, Propylene, Glycol, Xylitol, PEG‐8, PEG‐40‐Hydrogenated castor oil, Cocamidopropyl betaine, Dipotassium phosphate, Aroma, Sodium chloride, Cellulose gum, Rebaudioside A, Sodium fluoride (1′400 ppm), Diamond powder, CI 42090, Limonene.	Silica, Diamond powder

* REA and RDA values by Hamza et al. [16].

A slurry of each toothpaste was mixed in the ratio of one part toothpaste: two parts artificial saliva according to a recipe by Klimek et al. with a pH value of 6.4 [23].

Samples were brushed with 3 mL of the respective toothpaste slurry in a custom‐made automatic brushing machine with a frequency of 60 brushing cycles per minute with reciprocating movements at a constant brushing force of 2.5 N for 6 h. This results in a total of 21,600 brushing cycles per sample. The slurry was renewed every 1.5 h (every 5400 brushing cycles) without rinsing.

### 2.3 Measurement of Abrasive Enamel Wear

The abrasive enamel wear was measured profilometrically using a stylus profilometer (Perthometer S2, Mahr, Göttingen, Germany). Before starting the brushing procedure, five surface profiles per sample at a distance of 250 μm from each other for a total length of 4.8 mm perpendicular to the brushing direction were recorded. To ensure accurate repositioning, samples were equipped with a notch and the profilometer with a specially designed mounting device. Final measurements were taken after the brushing process. The abrasive enamel wear was then calculated by superimposing the profiles before and after brushing using customized software. The height differences between the before and after brushing profiles were interpreted as the abrasive enamel wear.

### 2.4 Statistical Analysis

Data were collected using Microsoft Excel (Microsoft Corporation, Redmond, Washington, USA) and statistical testing was conducted using the language and environment for statistical computing “R” [29, 30, 31, 32, 33, 34, 35, 36, 37, 38, 39, 40, 41, 42, 43, 44, 45, 46, 47, 48, 49, 50, 51, 52, 53, 54, 57, 58].

Mean values, standard deviations, median and IQR of the abrasive enamel wear for the two toothbrushes in combination with each of the four toothpastes were calculated. Wilcoxon signed‐rank tests were applied to investigate any significant differences between the two different toothbrushes within the toothpaste groups. Pairwise tests were conducted for comparisons between the different toothpastes within the toothbrush groups. *p*‐values were adjusted following the Holm method for multiple comparisons and the significance level was set at 0.05.

## 3 Results

Abrasive enamel wear after 6 h of brushing with the respective toothbrushes and toothpastes is presented in Table 4.

**TABLE 4:** Abrasive enamel wear in μm (median/IQR) for the respective combinations of bristle end configuration and toothpaste. Same letters indicate no statistically significant differences between the groups (both vertically and horizontally).

Toothpaste	Round‐end	Tapered‐end
Colgate total original	0.00/0.00	0.01/0.00
Candida white diamond	47.61/18.66	7.16/2.23
Swiss smile diamond glow	51.36/6.34	29.71/15.35
Emoform F diamond	16.91/2.86	5.27/2.11

### 3.1 Related to the Toothbrush

For both toothbrushes (round‐end and tapered‐end; median/IQR), SSDG caused the highest abrasive enamel wear (51.36/6.34 μm resp. 29.71/15.35 μm), followed by CWD (47.61/18.66 μm resp. 7.16/2.23 μm), EFD (16.91/2.86 μm resp. 5.27/2.11 μm) and CT (0.00/0.00 μm resp. 0.01/0.00 μm) with *p* < 0.05. (Table 4).

### 3.2 Related to the Toothpaste

Within all three diamond‐based toothpaste groups (CWD, SSDG and EFD), the round‐end toothbrush caused statistically significantly more abrasion than the tapered one (*p* < 0.05). For the silicate‐based toothpaste (CT), no statistically significant abrasive enamel wear could be measured for both toothbrushes.

## 4 Discussion

In studies involving dentine samples, bovine teeth were identified as a suitable alternative to human teeth for abrasion studies [25, 26, 27, 28, 29, 30, 31, 32, 33, 34, 35, 36, 37, 38, 39, 40, 41, 42, 43, 44, 45, 46]. For enamel, the existing literature seems to be more inconsistent [4, 5, 6, 7, 8, 9, 10, 11, 12, 13, 14, 15, 16, 17, 18, 19, 20, 21, 22, 23, 24, 25, 26, 27, 28, 29, 30, 31, 32, 33, 34, 35, 36, 37, 38, 39, 40, 41, 42, 43, 44, 45, 46, 47, 48, 49, 50, 51, 52, 53, 54, 57]. However, Wang et al. argued that mandibular bovine teeth could still be used as an alternative to human teeth for dental research due to their similarity in enamel microstructure [45].

In the preliminary tests of this study, the brushing part was designed to simulate approximately 8 years of manual tooth brushing. In order to prevent the complete removal of the enamel with highly abrasive diamond powder‐based toothpastes and thus falsify the results, brushing was performed for 6 h, i. e, 21′600 cycles, 43′200 brushing strokes, respectively. Following the recommendations to brush teeth two to three times a day, this represents an actual brushing time of approximately 4 years when following the assumptions of Wiegand and Attin of 10 brushing strokes per tooth per brushing session [48]. In addition, the reduction of brushing time should also prevent the formation of deep grooves in which the bristles would rest more frequently, thereby increasing the uneven abrasion of the surface.

The brushing force of 2.5 N was selected within the range of 2–3 N used for most dental abrasion studies [48].

The abrasive slurries used in this study were prepared from four different toothpastes. According to a classification model described by Imfeld et al. [22], the slurry with silicate particles (control group) is classified as low abrasive with an REA value of 4; the abrasive slurries of the toothpastes with diamond powder are classified as very strong abrasive with REA > 8 [16]. Toothpastes with diamond powder repeatedly showed very gentle abrasion values on dentine while at the same time behaving much more abrasive on enamel [9, 10, 11, 12, 13, 14, 15, 16, 17, 18, 19, 20, 21, 22]. The toothpastes examined in this study were selected to cover a wide range of REA values. Like the majority of traditional toothpastes, CT is based on silicates and was chosen as control because it has already been used in numerous other studies [9, 10, 11, 12, 13, 14, 15, 16, 17, 18, 19, 20, 21, 22, 23, 24, 25, 26, 27, 28, 29, 30, 31, 32, 33, 34, 35, 36, 37, 38, 39, 40, 41].

The specifications of the two types of specially made toothbrushes used in this study were kept identical to the biggest amount possible. However, for manufacturing reasons, the bristle designs differed in length by 0.5 mm, with a tolerance of 0.2 mm (Table 1 and Figure 1). It is considered that the tapered‐end bristles can be bent more easily under pressure when in contact with the tooth surface. Several studies have investigated the influence of bristle length and stiffness [6, 7, 8, 9, 10, 11, 12, 13, 14, 15, 16, 17, 18, 19]. In some cases, significant differences in outcomes have been found. On the other hand, bristle stiffness was also shown to only have a minor effect on substance loss in dentine and not in enamel [26]. Whether this slight difference in bristle length has an influence on the results would therefore need to be investigated further in detail.

**FIGURE 1:**
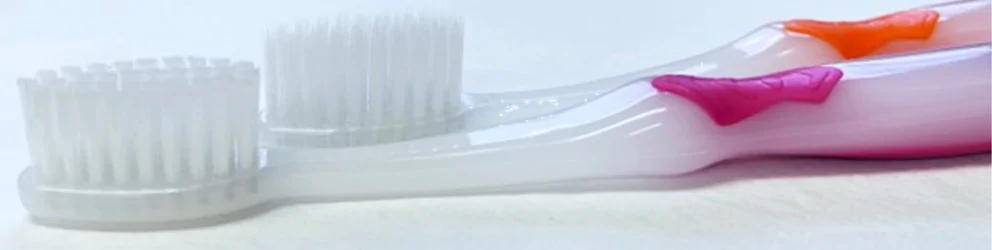
Toothbrushes used (left: Round‐end toothbrush; right: Tapered‐end toothbrush).

The measurement limit of the abrasive enamel measured by the profilometer and subsequently calculated was 0.105 μm [3, 4, 5, 6, 7, 8, 9, 10, 11, 12, 13, 14, 15, 16, 17, 18, 19, 20, 21, 22, 23, 24, 25, 26, 27, 28, 29, 30, 31, 32, 33, 34, 35, 36, 37, 38, 39, 40]. Therefore, the value for the loss of these profiles was set to 0.000 μm. Based on our experiments, it can be stated that for the only silicate‐based toothpaste (CT) no measurable ablation could be detected with the protocol used in this study.

The first null hypotheses, that there is no significant difference in the resulting enamel wear when using toothbrushes with round‐end or tapered‐end bristles has to be rejected as the two different bristle configurations used showed significantly different abrasive enamel wear in three out of four toothpastes. As the abrasive enamel wear using CT was below detection level, no significant difference could be found for this group. For all groups with toothpastes containing diamond powder, significantly higher enamel wear was observed when brushing with round bristle ends than with tapered bristle ends. Hamza et al. made the same observation for dentine [19]. One possible explanation could be that the tapered bristles have a tendency to bend more easily. There are several reasons for this. Due to the tapered ends, the diameter at the bristle end is inevitably smaller, so the tapered end is less rigid and also has more room to bend due to its reduced volume. The increased bending of the bristle tip could result in the abrasive particles being pushed across the tooth surface more often and for a longer period of time. This was observed on dentine in an abrasion study with soft toothbrushes [20]. It is discussed whether round bristle ends, on the other hand, hold particles better and can therefore grind harder on the tooth surface [19].

The second null hypotheses, that there would be no difference in the absolute abrasive enamel wear when using toothpastes with highly discrepant REA values, has to be rejected too as there were significant differences in the enamel wear when different toothpastes were used. Within the limitations of this study, it has been shown that the supposedly dentine preserving (low RDA) toothpastes with diamond powder [16, 17, 18, 19, 20, 21, 22] tend to be much more abrasive on enamel; this was also discussed by Hamza et al. The abrasive enamel wear of diamond particles was significantly higher than that of silicates. It could be assumed that this might be due to the relatively higher hardness of diamond particles compared to silicate particles. One could imagine that the diamond particles are scratched over the enamel surface by the bristles of the toothbrush in a highly abrasive manner and thus lead to relatively heavy wear, whereas they have less effect on the relatively softer dentine and tend to roll over the surface or be pressed into it. This study also confirms previous findings that the results of in vitro abrasion studies can give very different results on enamel and dentine [9, 10, 11, 12, 13, 14, 15, 16, 17, 18, 19, 20, 21, 22, 23, 24, 25, 26, 27, 28, 29, 30, 31, 32, 33, 34, 35, 36, 37, 38, 39, 40, 41, 42, 43, 44, 45, 46]. The results of this study support the statement of Dobler et al. that the REA value should be declared on the toothpaste in addition to the RDA value that is usually stated on the packaging [9].

As already established in other studies, absolute abrasive enamel wear did not correlate with the REA value [28, 29, 30, 31, 32, 33, 34, 35, 36, 37, 38, 39, 40, 41]. CWD has a higher REA value (244), but in this study its absolute enamel wear was significantly lower than that of SSD (177). This may be due to the fact that the two characteristics were measured under different conditions. Lippert et al. state that the RDA and REA values of the toothpastes were obtained under atypical in vivo conditions and do not correlate linearly with clinical or laboratory observations [26]. The RDA and REA values can provide an indication of abrasivity, but do not correlate with the effective, quantitative removal of tooth substance [14, 15, 16, 17, 18, 19, 20, 21, 22, 23, 24, 25, 26, 27, 28, 29, 30, 31, 32, 33, 34, 35, 36, 37, 38, 39, 40, 41]. Due to different composition of toothpastes and non‐linear correlation between REA and quantitative abrasion [28], it can be assumed that the results would be disparate if alternative toothpastes were used.

The preceding points serve to illustrate the necessity of considering a multitude of factors when making recommendations regarding oral hygiene products. These include the abrasive particles present in toothpaste, the bristle ends of the toothbrush, and numerous other variables that have not yet been investigated in detail. It is recommended that further variables and their interactions be investigated in future studies to determine their effects on enamel abrasion and to choose the best combination of applied toothpaste and toothbrush for effective cleaning with least possible hard tissue abrasion.

## 5 Conclusion

Within the limitations of the present study, it can be concluded that in combination with diamond‐based toothpastes, tapered‐end toothbrushes cause less abrasive enamel wear compared to round‐end toothbrushes.

## 6 Clinical Relevance

### 6.1 Scientific Rationale for Study

Recent studies have been focusing on the large variety of different aspects of toothbrushing contributing to abrasion. However—to the best of the authors knowledge—the influence of different toothbrush bristle configurations (round‐end vs. tapered‐end) on abrasive enamel wear has not yet been investigated, specifically with the use of newly introduced diamond powder‐based toothpastes, which were proven to be relatively highly abrasive on enamel.

### 6.2 Principal Findings

In combination with diamond powder‐based toothpaste, tapered‐end toothbrushes caused significantly less abrasive enamel wear compared to round‐end toothbrushes.

### 6.3 Practical Implications

The use of tapered‐end toothbrushes could offer a more enamel‐protecting choice for patients using diamond powder‐based toothbrushes.

## Funding

The authors have nothing to report.

## Conflicts of Interest

The authors declare no conflicts of interest.

## Data Availability

The data that support the findings of this study are available from the corresponding author upon reasonable request.

## References

[1] J. F. Alexander , A. J. Saffir , and W. Gold , “The Measurement of the Effect of Toothbrushes on Soft Tissue Abrasion,” Journal of Dental Research 56 (1977): 722–727, 10.1177/00220345770560070301. 269150

[2] G. Anneroth and A. Poppelman , “Histological Evaluation of Gingival Damage by Toothbrushing. An Experimental Study in Dog,” Acta Odontologica Scandinavica 33 (1975): 119–127, 10.3109/00016357509026352. 1067715

[3] T. Attin , K. Becker , M. Roos , R. Attin , and F. Paqué , “Impact of Storage Conditions on Profilometry of Eroded Dental Hard Tissue,” Clinical Oral Investigations 13 (2009): 473–478, 10.1007/s00784-009-0253-9. 19214602

[4] T. Attin , F. Wegehaupt , D. Gries , and A. Wiegand , “The Potential of Deciduous and Permanent Bovine Enamel as Substitute for Deciduous and Permanent Human Enamel: Erosion‐Abrasion Experiments,” Journal of Dentistry 35 (2007): 773–777, 10.1016/j.jdent.2007.07.007. 17709163

[5] C. M. Barnes , D. A. Covey , X. Shi , and S. L. Yankell , “Laboratory Evaluations of a Bi‐Level, Extremely Tapered Bristled Toothbrush and a Conventional Uniform Bristled Toothbrush,” American Journal of Dentistry 22 (2009): 84–88. 19626970

[6] M. Bizhang , K. Riemer , W. H. Arnold , J. Domin , and S. Zimmer , “Influence of Bristle Stiffness of Manual Toothbrushes on Eroded and Sound Human Dentin ‐ An in Vitro Study,” PLoS One 11 (2016): e0153250, 10.1371/journal.pone.0153250. PMC4829200 27070901

[7] M. Bizhang , I. Schmidt , Y. P. Chun , W. H. Arnold , and S. Zimmer , “Toothbrush Abrasivity in a Long‐Term Simulation on Human Dentin Depends on Brushing Mode and Bristle Arrangement,” PLoS One 12 (2017): e0172060, 10.1371/journal.pone.0172060. PMC5319671 28222156

[8] J. Breitenmoser , W. Mörmann , and H. R. Mühlemann , “Damaging Effects of Toothbrush Bristle End Form on Gingiva,” Journal of Periodontology 50 (1979): 212–216, 10.1902/jop.1979.50.4.212. 374707

[9] L. Dobler , B. Hamza , T. Attin , and F. J. Wegehaupt , “Abrasive Enamel and Dentin Wear Resulting From Brushing With Toothpastes With Highly Discrepant Relative Enamel Abrasivity (REA) and Relative Dentin Abrasivity (RDA) Values,” Oral Health & Preventive Dentistry 21 (2023): 41–48, 10.3290/j.ohpd.b3858625. PMC11619904 36727837

[10] C. E. Dörfer , E. R. von Bethlenfalvy , B. Kugel , and T. Pioch , “Cleaning Efficacy of a Manual Toothbrush With Tapered Filaments,” Oral Health & Preventive Dentistry 1 (2003): 111–118. 15645932

[11] J. Gallob , D. M. Petrone , L. R. Mateo , et al., “Comparative Efficacy of a Soft Toothbrush With Tapered‐Tip Bristles to an ADA Reference Toothbrush on Gingival Abrasion Over a 12‐Week Period,” Journal of Clinical Dentistry 27 (2016): 48–53. 28390206

[12] J. Gallob , D. M. Petrone , L. R. Mateo , et al., “Comparative Efficacy of a Soft Toothbrush With Tapered‐Tip Bristles and an ADA Reference Toothbrush on Established Gingivitis and Supragingival Plaque Over a 12‐Week Period,” Journal of Clinical Dentistry 27 (2016): 39–47. 28390205

[13] A. Ghassemi , L. Vorwerk , W. Hooper , et al., “Comparative Plaque Removal Efficacy of Three Manual Toothbrushes,” Journal of Clinical Dentistry 27 (2016): 71–75. 28390200

[14] C. González‐Cabezas , A. T. Hara , J. Hefferren , and F. Lippert , “Abrasivity Testing of Dentifrices ‐ Challenges and Current State of the Art,” Monographs in Oral Science 23 (2013): 100–107, 10.1159/000350476. 23817063

[15] B. Hamza , A. Abdulahad , T. Attin , and F. J. Wegehaupt , “Diamond Particles in Toothpastes: In‐Vitro Effect on the Abrasive Enamel Wear,” BMC Oral Health 22 (2022): 248, 10.1186/s12903-022-02274-3. PMC9219232 35733139

[16] B. Hamza , T. Attin , C. Cucuzza , A. Gubler , and F. J. Wegehaupt , “RDA and REA Values of Commercially Available Toothpastes Utilising Diamond Powder and Traditional Abrasives,” Oral Health & Preventive Dentistry 18 (2020): 807–814, 10.3290/j.ohpd.a45085. PMC11654561 32895665

[17] B. Hamza , T. Attin , P. N. Paqué , D. B. Wiedemeier , and F. J. Wegehaupt , “Mechanical Properties of Toothpastes With Diamond Abrasives,” Journal of Esthetic and Restorative Dentistry 32 (2020): 416–423, 10.1111/jerd.12588. 32277866

[18] B. Hamza , M. Niedzwiecki , P. Körner , T. Attin , and F. J. Wegehaupt , “Effect of the Toothbrush Tuft Arrangement and Bristle Stiffness on the Abrasive Dentin Wear,” Scientific Reports 12 (2022): 840, 10.1038/s41598-022-04884-x. PMC8764029 35039599

[19] B. Hamza , L. Svellenti , P. Körner , T. Attin , and F. J. Wegehaupt , “Effect of Tapered‐End and Round‐End Bristles on the Abrasive Dentine Wear Applying Increasing Brushing Forces,” Acta Odontologica Scandinavica 80, no. 6 (2022): 465–469, 10.1080/00016357.2022.2035816. 35147472

[20] B. Hamza , M. Tanner , P. Körner , T. Attin , and F. J. Wegehaupt , “Effect of Toothbrush Bristle Stiffness and Toothbrushing Force on the Abrasive Dentine Wear,” International Journal of Dental Hygiene 19 (2021): 355–359, 10.1111/idh.12536. PMC8597153 34270163

[21] E. Hellwig , E. Schäfer , J. Klimek , and T. Attin , Einführung in die Zahnerhaltung (Deutscher Zahnärzte Verlag, 2018), 699–716.

[22] T. Imfeld , B. Sener , and F. Lutz , “Mechanische Wirkung von in der Schweiz marktführenden Zahnpasten auf Dentin,” Acta Medicae Dentalis Helveticae 3 (1998): 354–359.

[23] J. Klimek , E. Hellwig , and G. Ahrens , “Fluoride Taken Up by Plaque, by the Underlying Enamel and by Clean Enamel From Three Fluoride Compounds in Vitro,” Caries Research 16 (1982): 156–161, 10.1159/000260592. 6951638

[24] G. P. J. Langa , F. W. M. G. Muniz , T. P. Wagner , C. F. E. Silva , and C. K. Rösing , “Anti‐Plaque and Anti‐Gingivitis Efficacy of Different Bristle Sitffness and End‐Shape Toothbrushes on Interproximal Surfaces: A Systematic Review With Meta‐Analysis,” Journal of Evidence‐Based Dental Practice 21 (2021): 101548, 10.1016/j.jebdp.2021.101548. 34391550

[25] P. Laurance‐Young , L. Bozec , L. Gracia , et al., “A Review of the Structure of Human and Bovine Dental Hard Tissues and Their Physicochemical Behaviour in Relation to Erosive Challenge and Remineralisation,” Journal of Dentistry 39 (2011): 266–272, 10.1016/j.jdent.2011.01.008. 21277346

[26] F. Lippert , M. A. Arrageg , G. J. Eckert , and A. T. Hara , “Interaction Between Toothpaste Abrasivity and Toothbrush Filament Stiffness on the Development of Erosive/Abrasive Lesions in Vitro,” International Dental Journal 67 (2017): 344–350, 10.1111/idj.12305. PMC9378893 28574173

[27] J. L. B. Maciel , R. Geng Vivanco , and F. C. P. Pires‐de‐Souza , “Remineralization, Color Stability and Surface Roughness of Tooth Enamel Brushed With Activated Charcoal‐Based Products,” Journal of Esthetic and Restorative Dentistry 35 (2023): 1144–1151, 10.1111/jerd.13057. 37083113

[28] C. J. Philpotts , E. Weader , and A. Joiner , “The Measurement in Vitro of Enamel and Dentine Wear by Toothpastes of Different Abrasivity,” International Dental Journal 55 (2005): 183–187, 10.1111/j.1875-595x.2005.tb00057.x. 16004251

[29] T. Pohlert , “The pairwise multiple comparison of mean ranks package (PMCMR),” (2014), https://CRAN.R‐project.org/package=PMCMR.

[30] T. Pohlert , “PMCMRplus: Calculate pairwise multiple comparisons of mean rank sums extended,” (2023).

[31] R. J. Radlanski , Orale Struktur‐ und Entwicklungsbiologie (Quintessenz Verlags‐GmbH, 2011), 606–618.

[32] H. Rahman , “Comparison of Manual Toothbrushes With Different Bristle Designs in Terms of Cleaning Efficacy and Plaque Control: A Pilot Study,” Indian Journal of Community Medicine 48 (2023): 108–111, 10.4103/ijcm.ijcm_404_22. PMC10112751 37082387

[33] N. Ranzan , F. W. M. G. Muniz , and C. K. Rösing , “Are Bristle Stiffness and Bristle End‐Shape Related to Adverse Effects on Soft Tissues During Toothbrushing? A Systematic Review,” International Dental Journal 69 (2019): 171–182, 10.1111/idj.12421. PMC9379007 30152076

[34] N. A. Rosema , M. F. Timmerman , P. A. Versteeg , W. H. Van Palenstein Helderman , U. Van Der Velden , and G. A. Van Der Weijden , “Safety and Efficacy of Two Manual Toothbrushes,” International Journal of Dental Hygiene 8 (2010): 280–285, 10.1111/j.1601-5037.2010.00475.x. 20961384

[35] G. Sangnes and P. Gjermo , “Prevalence of Oral Soft and Hard Tissue Lesions Related to Mechanical Toothcleansing Procedures,” Community Dentistry and Oral Epidemiology 4 (1976): 77–83, 10.1111/j.1600-0528.1976.tb01607.x. 1062255

[36] U. P. Saxer , T. Nittner , and H. Toutenburg , “Acceptance of the Meridol Toothbrush by Dental Hygienists. A Questionnaire Study,” Schweizerische Monatsschrift Fur Zahnmedizin 117 (2007): 1050–1058. 17987879

[37] H. D. Sgan‐Cohen , A. Livny , and Y. Vered , “The Elmex SENSITIVE Toothbrush: Effect on Plaque Reduction and Subjective Satisfaction After Two Months,” Journal of Clinical Dentistry 19 (2008): 22–27. 18500156

[38] H. D. Sgan‐Cohen and Y. Vered , “Plaque Removal and Oral Health Promotion Potential for the Elmex interX Medium Toothbrush: Clinical Efficacy and Safety Evaluation,” Journal of Clinical Dentistry 14 (2003): 70–73. 14520777

[39] H. D. Sgan‐Cohen and Y. Vered , “A Clinical Trial of the Meridol Toothbrush With Conical Filaments: Evaluation of Clinical Effectiveness and Subjective Satisfaction,” Journal of Clinical Dentistry 16 (2005): 109–113. 16583594

[40] V. P. Shah , K. K. Midha , J. W. Findlay , et al., “Bioanalytical Method Validation ‐ a Revisit With a Decade of Progress,” Pharmaceutical Research 17 (2000): 1551–1557, 10.1023/a:1007669411738. 11303967

[41] P. N. Tawakoli , K. Becker , and T. Attin , “Abrasive Effects of Diamond Dentifrices on Dentine and Enamel,” Swiss Dent J 128 (2018): 14–19, 10.61872/sdj-2018-01-365. 29533050

[42] P. N. Tawakoli , B. Sener , and T. Attin , “Mechanical Effects of Different Swiss Market‐Leading Dentifrices on Dentin,” Swiss Dental Journal 125 (2015): 1210–1219, 10.61872/sdj-2015-11-159. 26631278

[43] Team RC , “R: A language and environment for statistical computing,” (2015), https://www.R‐project.org/.

[44] G. T. Terezhalmy , S. A. Bsoul , R. D. Bartizek , and A. R. Biesbrock , “Plaque Removal Efficacy of a Prototype Manual Toothbrush Versus an ADA Reference Manual Toothbrush With and Without Dental Floss,” Journal of Contemporary Dental Practice 6 (2005): 1–13. 16127467

[45] C. Wang , Y. Fang , L. Zhang , Z. Su , J. Xu , and B. Fu , “Enamel Microstructural Features of Bovine and Human Incisors: A Comparative Study,” Annals of Anatomy 235 (2021): 151700, 10.1016/j.aanat.2021.151700. 33588042

[46] F. J. Wegehaupt , R. Widmer , and T. Attin , “Is Bovine Dentine an Appropriate Substitute in Abrasion Studies?,” Clinical Oral Investigations 14 (2010): 201–205, 10.1007/s00784-009-0283-3. 19452177

[47] H. Wickham , “tidyverse: Easily install and load the ‚Tidyverse,” (2017), https://CRAN.R‐project.org/package=tidyverse.

[48] A. Wiegand and T. Attin , “Design of Erosion/Abrasion Studies ‐ Insights and Rational Concepts,” Caries Research 45, no. Suppl 1 (2011): 53–59, 10.1159/000325946. 21625133

[49] A. Wiegand and N. Schlueter , “The Role of Oral Hygiene: Does Toothbrushing Harm?,” Monographs in Oral Science 25 (2014): 215–219, 10.1159/000360379. 24993269

[50] S. L. Yankell , X. Shi , and R. C. Emling , “Laboratory Interproximal Access Efficacy and Gingival Margin Cleaning of the Elmex SENSITIVE SOFT, EXTRA SOFT and ADA Reference Toothbrushes,” Journal of Clinical Dentistry 18 (2007): 25–28. 17410953

[51] S. L. Yankell , C. M. Barnes , X. Shi , and J. Cwik , “Laboratory Efficacy of Three Compact Toothbrushes to Reduce Artificial Plaque in Hard to Reach Areas,” American Journal of Dentistry 24 (2011): 195–199. 22016911

[52] S. L. Yankell , X. Shi , and R. C. Emling , “Laboratory Evaluations of Two Toothbrushes for Removal of Artificial Plaque Above, Around and Below the Gingival Margin,” Journal of Clinical Dentistry 14 (2003): 19–22. 12619266

[53] S. L. Yankell , X. Shi , R. C. Emling , and H. C. Kang , “Comparing the Dr. SEDOC and ADA Toothbrushes in Three Independent Laboratory Procedures,” Journal of Clinical Dentistry 15 (2004): 33–36. 15218714

[54] S. L. Yankell , X. Shi , R. C. Emling , C. M. Spirgel , and P. A. Scheier , “Development of the Evolve Toothbrush: Laboratory Evaluations of Gingival Margin Cleaning and Subgingival Access,” Journal of Clinical Dentistry 18 (2007): 91–94. 17913004

[55] M. Addy , “Oral Hygiene Products: Potential for Harm to Oral and Systemic Health,” Periodontology 2000 48 (2008): 54–65, 10.1111/j.1600-0757.2008.00253.x. 18715356

[56] M. Addy and M. L. Hunter , “Can Tooth Brushing Damage Your Health? Effects on Oral and Dental Tissues,” International Dental Journal 53, no. 3 (2003): 177–186, 10.1111/j.1875-595x.2003.tb00768.x. 12875306

[57] G. H. Yassen , J. A. Platt , and A. T. Hara , “Bovine Teeth as Substitute for Human Teeth in Dental Research: A Review of Literature,” Journal of Oral Science 53 (2011): 273–282, 10.2334/josnusd.53.273. 21959653

[58] H. Zhu , “kableExtra: Construct Complex Table with ‘kable’ and Pipe Syntax,” (2021).

